# Supplementary material for: Yeast Translation Elongation Factor eIF5A Expression Is Regulated by Nutrient Availability through Different Signalling Pathways
Source: Int J Mol Sci. 2020 Dec 28;22(1):219. doi: 10.3390/ijms22010219 (PMC7794953; doi:10.3390/ijms22010219)
Supplement: Supplementary file 1 [file ijms-22-00219-s001.pdf]

Table S1. Yeast strains used in this study

Name	Genotype	Source
**BY4741**	MATa *ura3Δ0 leu2Δ0 his3Δ1 met15Δ0*	Euroscarf
hap1Δ	BY4741 MATa *ura3Δ0leu2Δ0 his3Δ1 met15Δ0 YLR256w::kanMX6*	This study
hap4Δ	BY4741 MATa *ura3Δ0 leu2Δ0 his3Δ1 met15Δ0 YKL109w::kanMX4*	Euroscarf
lia1Δ	BY4741 MATa *ura3Δ0 leu2Δ0 his3Δ1 met15Δ0 YJR070c::kanMX4*	Euroscarf
mpc1Δ	BY4741 MATa *ura3Δ0 leu2Δ0 his3Δ1 met15Δ0 YGL080w::kanMX4*	Euroscarf
msn2Δ	BY4741 MATa *ura3Δ0 leu2Δ0 his3Δ1 met15Δ0 YMR037c::kanMX4*	Euroscarf
pda1Δ	BY4741 MATa *ura3Δ0 leu2Δ0 his3Δ1 met15Δ0 YER178w::kanMX4*	Euroscarf
snf1Δ	BY4741 MATa *ura3Δ0 leu2Δ0 his3Δ1 met15Δ0 YDR073w::kanMX4*	Euroscarf
tif51A-1	BY4741 MATa *ura3Δ0leu2Δ0 his3Δ1 met15Δ0 tif51A-1::kanR*	(Li *et al.* 2011)
tif51A-3	BY4741 MATa *ura3Δ0leu2Δ0 his3Δ1 met15Δ0 tif51A-3::kanR*	(Li *et al.* 2011)

Table S2. Oligonucleotides used in this study

Primer	Sequence (5'-3')
**Gene expression detection by RT-qPCR**	
ACT-F	TCGTTCCAATTTACGCTGGTT
ACT-R	CGGCCAAATCGATTCTCAA
CYC1-F	AGATGTCTACAATGCCACACC
CYC1-R	CCCTTCAGCTTGACCAGAGT
eIF2A-F	ATTCTACTCCGGCCCCATCT
eIF2A-R	TCTAGTTTGTCACCGACGGC
HAP1-F	TTGGACCTTCCTCACGAATC
HAP1-R	CAGGATCTTCACTGCCCATT
SDH1-F	CTCCAAGTTGACTTTGCTCAGAA
SDH1-R	ACGCGGAACCGTTTACAGA
TIF51-1	TCGACAATCTTACATGGTCT
TIF51A-1	CGATTCTACTTCTGTAGCCA
TIF51B-1	CTACACTTTAGTTCCCTTAC
**Gene disruption by PCR**	
HAP1-F1	GAAATAGAAGAAAAAGAAAAAAAAAAAAAAGGGAACAATAGGTTAGCGGATCCCCGGGTTAATTAA
HAP1-R1	TTACATTATCAATCCTTGCGTTTCAGCTTCCACTAATTTAGATGAGAATTCGAGCTCGTTTAAAC

Supplementary Figures

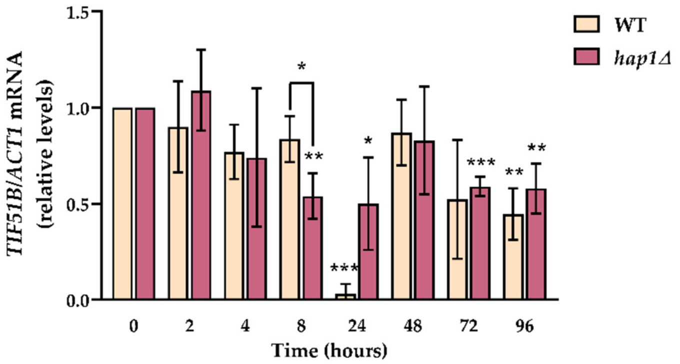

Supplementary Figure S1. Metabolic switch from fermentation to mitochondrial respiration yields a rapid drop of eIF5A but later recovery. WT and *hap1Δ* cells were grown in YPD medium during 96 hours and samples were collected at indicated time points. Relative *TIF51B* mRNA levels were determined. Results are shown as means±S.D. from three independent experiments and expressed relative to the value at time 0. Statistical significance was measured by Student's t-test relative to time 0. *$p < 0.05$, **$p < 0.01$, ***$p < 0.001$.

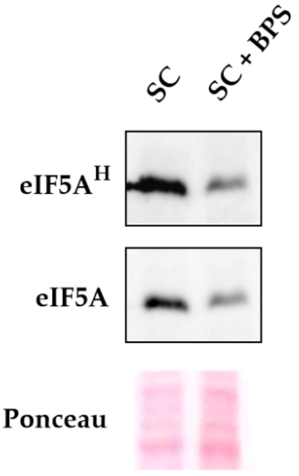

Supplementary Figure S2. Western blotting of eIF5A and hypusinated eIF5A in WT cells cultured in SC Complete medium with or without the addition of BPS (100 μM) for six hours. Red ponceau was used as loading control.

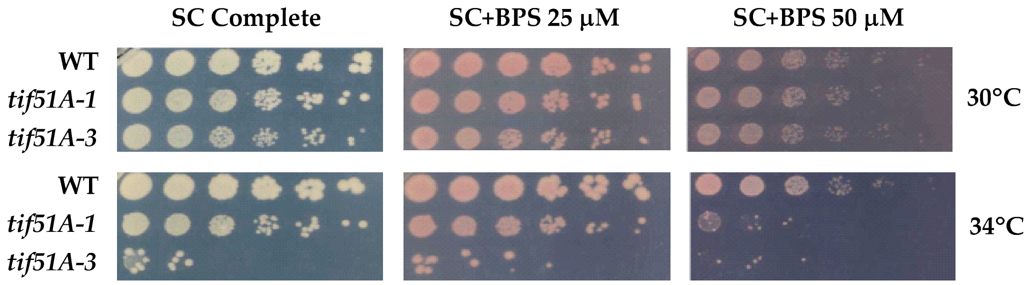

Supplementary Figure S3. Growth of the WT, *tif51A-1* and *tif51A-3* strains was tested in SC Complete medium with or without the addition of BPS at 25 µM or 50 µM at the indicated temperatures.

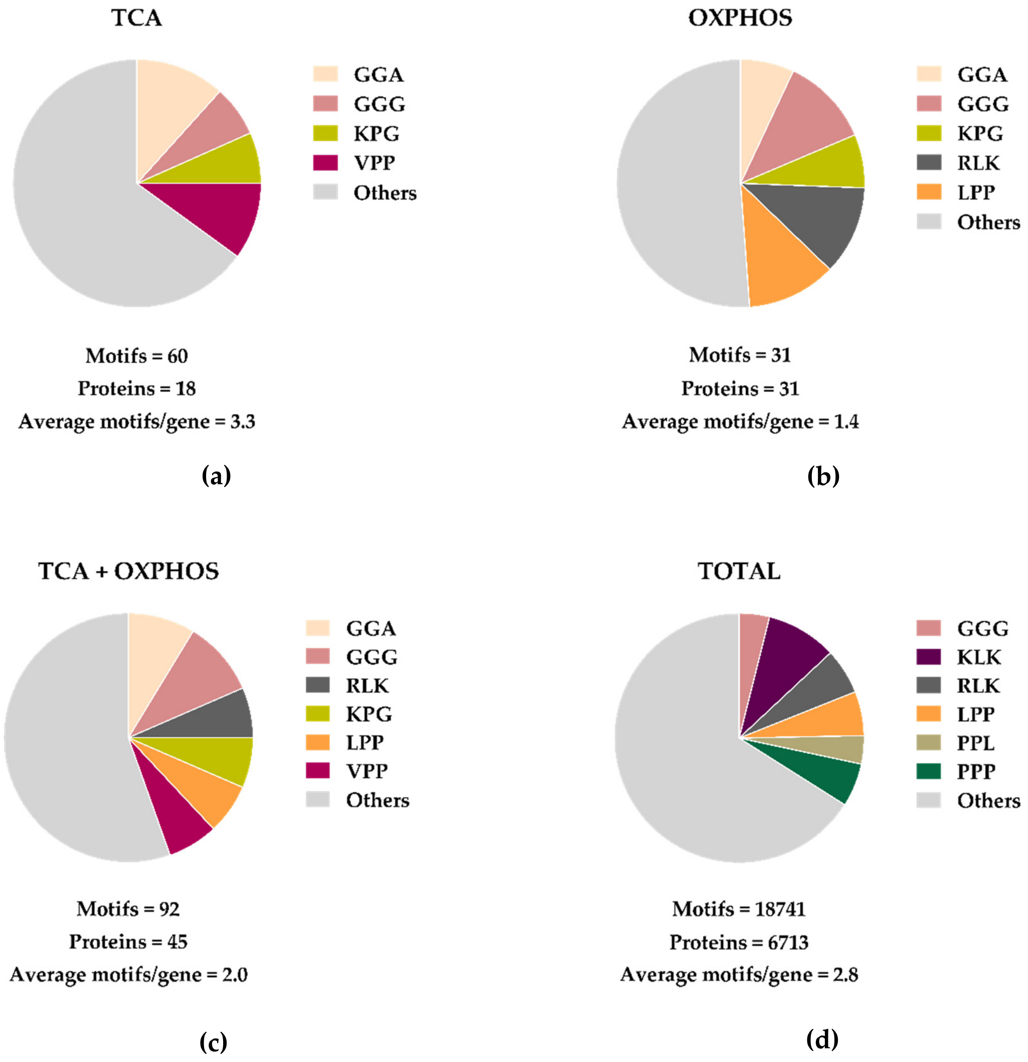

Supplementary Figure S4. Distribution of eIF5A-dependent motifsin the proteins of TCA and OXPHOS. Distribution of the 43-highest score eIF5A-dependent ribosome pausing motifs [2] in the proteins of TCA (a), OXPHOS (b), TCA + OXPHOS (c) and total proteins of yeast genome (d).
